# Characteristics of Diarrheal Illnesses in Non-Breast Fed Infants Attending a Large Urban Diarrheal Disease Hospital in Bangladesh

**DOI:** 10.1371/journal.pone.0058228

**Published:** 2013-03-08

**Authors:** Sanjoy Kumer Dey, Mohammod Jobayer Chisti, Sumon Kumar Das, Chandan Kumar Shaha, Farzana Ferdous, Fahmida Dil Farzana, Shahnawaz Ahmed, Mohammad Abdul Malek, Abu Syed Golam Faruque, Tahmeed Ahmed, Mohammed Abdus Salam

**Affiliations:** 1 Department of Neonatology, Bangabandhu Sheikh Mujib Medical University (BSMMU), Dhaka, Bangladesh; 2 Centre for Nutrition and Food Security (CNFS), International Centre for Diarrhoeal Disease Research, Bangladesh (icddr,b), Dhaka, Bangladesh; 3 Research Administration Services (RAS), icddr,b, Dhaka, Bangladesh; National Taiwan University Hospital, Taiwan

## Abstract

**Background:**

Lack of breast feeding is associated with higher morbidity and case-fatality from both bacterial and viral etiologic diarrheas. However, there is very limited data on the characteristics of non–breastfed infants attending hospital with diarrheal illnesses caused by common bacterial and viral pathogens. Our objective was to assess the impact of lack of breast feeding on diarrheal illnesses in infants living in urban Bangladesh.

**Methods:**

We extracted data of infants (0–11 months) for analyses from the data archive of Diarrheal Disease Surveillance System (DDSS) of the Dhaka Hospital of icddr,b for the period 2008–2011.

**Results:**

The prevalence of breastfeeding in infants attending the hospital with diarrhea reduced from 31% in 2008 to 17% in 2011, with corresponding increase in the prevalence of non-breastfed (chi square for trend <0.001). Among breastfed infants, the incidence of rotavirus infections was higher (43%) among the 0–5 months age group than infants aged 9–11 months (18%). On the other hand, among non-breastfed infants, the incidence of rotavirus infections was much higher (82%) among 9–11 months old infants compared to those in 0–5 months age group (57%) (chi square for trend <0.001). Very similar trends were also observed in the incidence of cholera and ETEC diarrheas among different age groups of breastfed and non-breastfed infants (chi square for trend 0.020 and 0.001 respectively). However, for shigellosis, the statistical difference remained unchanged among both the groups (chi square for trend 0.240).

**Conclusion and Significance:**

We observed protective role of breastfeeding in infantile diarrhea caused by the major viral and common bacterial agents. These findings underscore the importance of promotion and expansion of breastfeeding campaigns in Bangladesh and elsewhere.

## Introduction

In developing countries, diarrheal diseases, as a group, remains the leading cause of illness and the second leading cause of death among young children, particularly during the first two years of their lives. Diarrheal diseases accounted for 11% of the estimated 7.6 million under-five deaths, globally, in 2010 [1]. In Bangladesh, 11% of all under-five deaths (n = 182,936) was due to diarrhea [2]. Guidelines for the management of diarrheal illnesses have been refined, and new strategies for prevention and control of this major health problem have also been developed [3]. In developing countries, children are exposed to a wide range of enteric bacterial pathogens even at a very early age and suffer from frequent episodes of diarrheal illnesses. The distribution of predominant bacterial pathogens varies with the age of the children, which also vary by geographic locations as well as demonstrates seasonal and secular trends. The diarrheagenic *Escherichia coli, Shigella* spp., *Campylobacter* spp., and *Vibrio* are the common bacterial pathogens causing diarrhea in young children in developing countries, including Bangladesh [3]. Globally, rotavirus is one of the most common causes of dehydrating gastroenteritis among children younger than two years, which accounts for one-third of all hospitalizations for diarrhea and an estimated 500,000 deaths each year [4]. Compared to industrialized countries, children in the developing countries experience their first severe infection with rotavirus at a younger age, which follows distinct seasonal pattern, with winter peak, and they often are infected with multiple or un-typable strains of rotavirus [5]. The relationship between lack of breast feeding and diarrhea is well established [6], [7], [8], [9], [10], [11]. Non- breastfeeding is also associated with malnutrition, and diarrhea among this population caused by common enteric pathogens with higher risks for deaths [12], [13].

The human-milk glycans, which include oligosaccharides in their free and conjugated forms, constitute a major and an innate immunologic mechanism by which human milk protects breast-fed infants against infections [10]. Although there is increased overall literacy rate as well as emphasis has been given on early initiation and continued breastfeeding, sustained, high level of continued breastfeeding has not been achieved. There is limited recent information on the etiology of infantile diarrhea and distribution of pathogens by breastfeeding status of the infants. We, therefore, used a large database to analyze distribution of breastfeeding status among infants attending a large diarrheal disease treatment facility in Bangladesh, and examined distribution of diarrhoeal pathogen among breastfed and non-breastfed infants as well as by their age groups.

## Materials and Methods

### Ethical Statement

The Diarrhoeal Disease Surveillance System of icddr,b is a routine activity of the Dhaka Hospital, which has been approved by the Research Review Committee (RRC) and Ethical Review Committee (ERC) of icddr,b and this study has been approved by the RRC and ERC of icddr,b. Verbal consent was taken from the caregivers, or guardians on the behalf of the patients, for their information to be stored in the hospital database and used for research. Although the Diarrhoeal Disease Surveillance System of icddr,b is a routine activity for hospital patients, and used to be done only with verbal consent from the parents or guardians of the patients following the hospital policy, parents or guardians were assured about the non-disclosure of information collected from them, and were also informed about the use of data for analysis and using the results for improving patient care activities as well as publication without disclosing the name or identity of their children. ERC was quite satisfied with voluntary participation, the maintenance of the rights of the participants and confidential handling of personal information by the hospital physicians and approved this consent procedure.

### Study Population and Site

The study was conducted at the Dhaka Hospital of icddr,b. Located in the Dhaka, the capital city of Bangladesh, the hospital provides care and treatment to around 140,000 patients each year, vast majority of whom are under five children. Diarrhea is the common feature among the patients, whether or not they are associated with diarrhea-related complications or other health problems, while malnutrition is a common health problem in under-five children. The hospital also conducts research on enteric infections caused by common pathogens including rotavirus, ETEC, *V. cholerae*, and *Shigella*; acute respiratory infections; and malnutrition; and provides training to health care providers on case management and research methodology. Infants (0–11 months old), constitute about 28% of all attending patients. The majority of the patients come from poor socio-economic backgrounds, who live in urban and peri urban Dhaka.

### Study Design

For this descriptive study, we stratified infants by their etiologic agents then compared them by their breastfeeding status. Infants receiving any amount of breast milk at their current episode of diarrhea were defined as breast-fed and those not receiving any amount of breast milk constituted non-breast-fed infants in our analyses. Age stratification was done taking into consideration that exclusive breastfeeding should be continued till the end of age as 0–5 months (recommended age of exclusive breastfeeding), 6–8 months are the time when most of the infants receive their complementary feeding (beginning of the weaning food), and 9–11 months (post supplementary feeding when infants gradually switch to their family food).

### Source of Data and Hospital Surveillance System

icddr,b maintains a Diarrheal Disease Surveillance System (DDSS) at its Dhaka Hospital since 1979 [14]. The system currently enrolls a systematic 2% sample of all patients (every 50^th^ patient, regardless of age, sex and diarrhea severity) and records their socio-demographic and environmental history; nutritional and clinical characteristics; immunization status and feeding practices (this data is collected for only those younger than 3 years); hygienic practices in the family; and determines the etiology of diarrhea along with antimicrobial susceptibility of common bacterial pathogens. Trained research assistants interview patients (patients or a female caregiver in the event of minors) using a standardized questionnaire, performs anthropometry using standard techniques, and records findings in pre-tested case report forms (CRFs). A physician obtains medical history, performs thorough physical examination, and records findings in the CRFs.

### Specimen Collection and Laboratory Procedure

A fresh stool specimen is routinely collected from the surveillance patients for screening of common enteric pathogens such as rotavirus [15]; ETEC ([16]), *Vibrio cholerae* [17], and *Shigella* spp. [17] by standard methods.

### Data Analysis

We extracted relevant information from the electronic database of the Diarrheal Disease Surveillance System for our analyses for the period 2008–2011. In total, 2,869 infants (aged 0–11 months) of both sex, attended the hospital during this study period.

We used Statistical Package for Social Sciences (SPSS) Windows (Version 15.2; Chicago, IL) for data entry and analysis. We calculated the proportion of both breastfed and non-breastfed infants infected with enteropathogens (rotavirus, ETEC, *V. cholerae*, and *Shigella*) causing diarrhea with a view to describe their hospital visit patterns due to diarrheal illnesses in a large urban diarrheal disease hospital in Bangladesh. We have also calculated age stratified proportion of diarrhea pathogens among the breastfed and non-breastfed infants and yearly distribution of different diarrhea pathogens among the breastfed infants. We used Chi-square for trend to test statistical significance of the changing trend by year and age [18] and the assumptions were; the sample was random, each observation was classified into one cell only, and sample size was sufficiently large with adequate cell counts, 5 or more in all cells [19]. A probability of less than 0.05 was considered statistically significant. Strength of association was determined by calculating odds ratio (OR) and their 95% confidence intervals (CIs).

## Results

**Figure 1:**
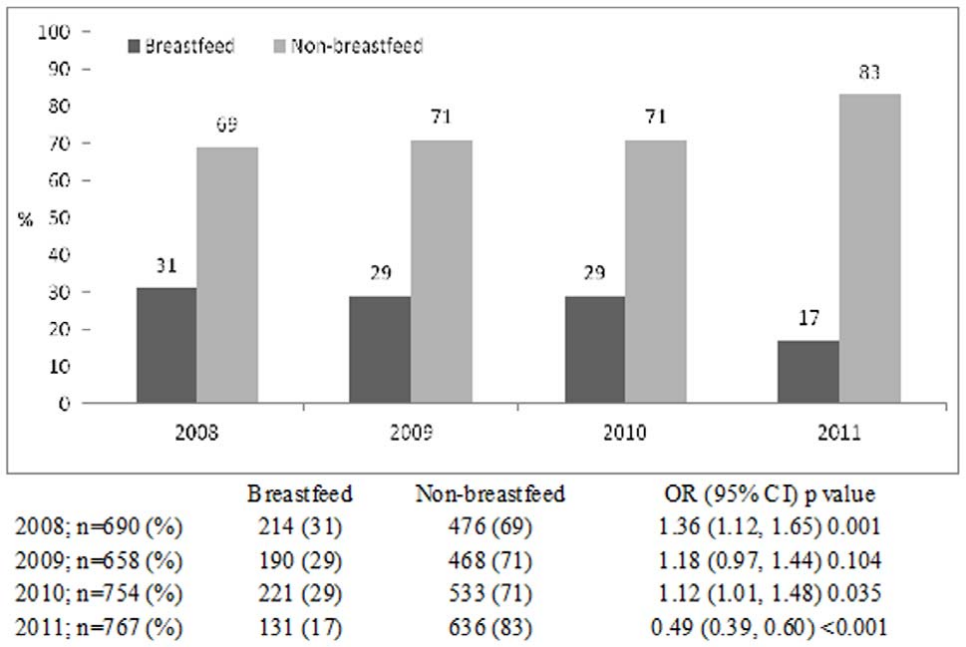
Yearly distribution of proportion of breastfeeding status of infants attending the hospital with diarrhea during 2008–11.

**Figure 2:**
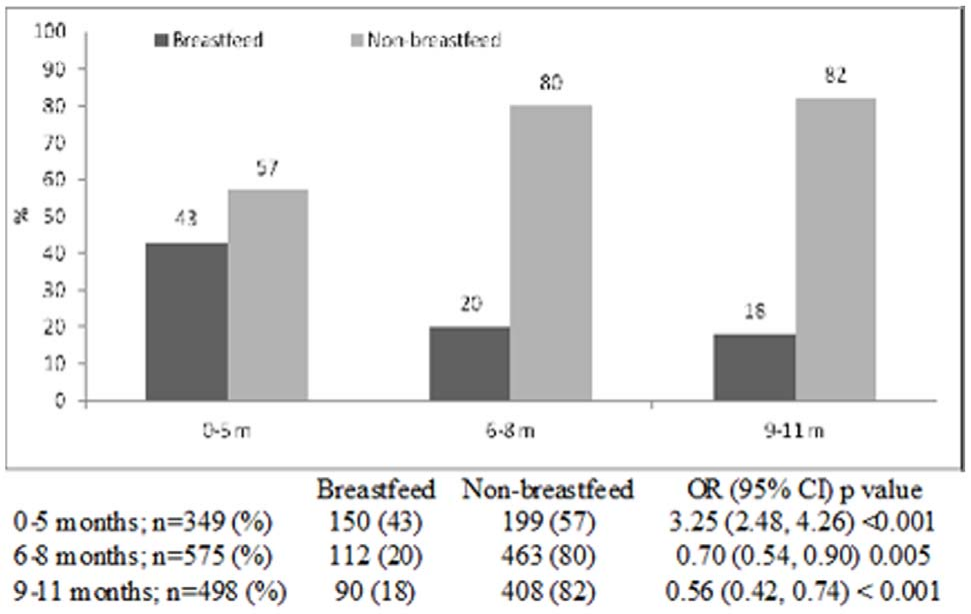
Age stratified proportion of rotavirus among breastfed and non-breastfed infants presented in the Dhaka Hospital, 2008–11.

**Figure 3:**
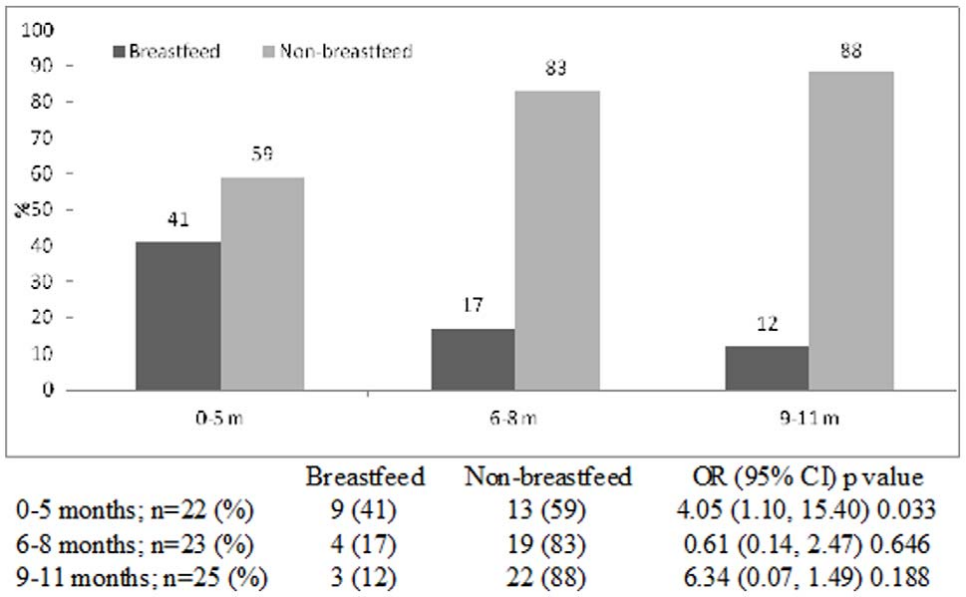
Age stratified proportion of *Vibrio cholerae* O1 among breastfed and non-breastfed infants presented in the Dhaka Hospital, 2008–11.

**Figure 4:**
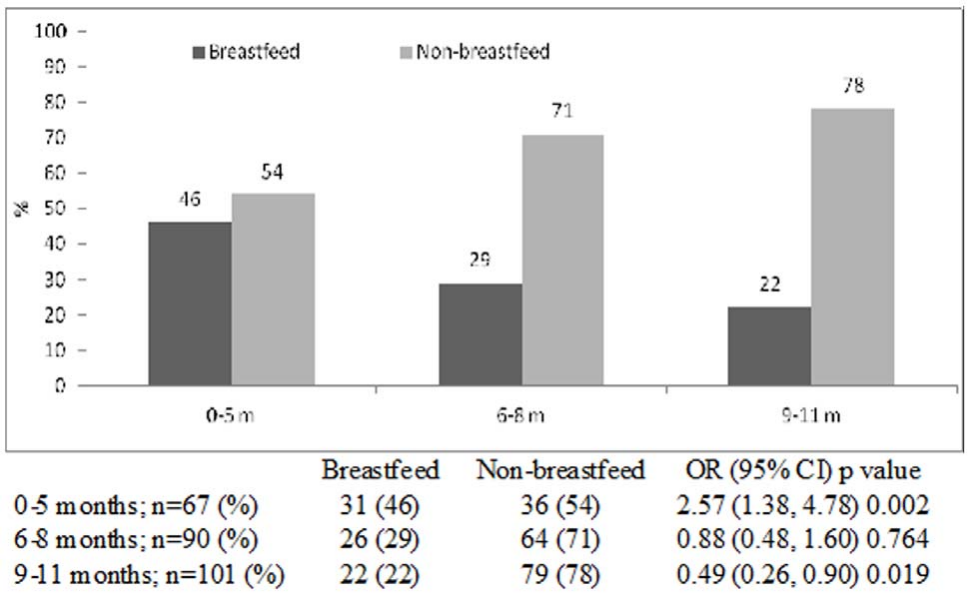
Age stratified proportion of ETEC among breastfed and non-breastfed infants presented in the Dhaka Hospital, 2008–11.

**Figure 5:**
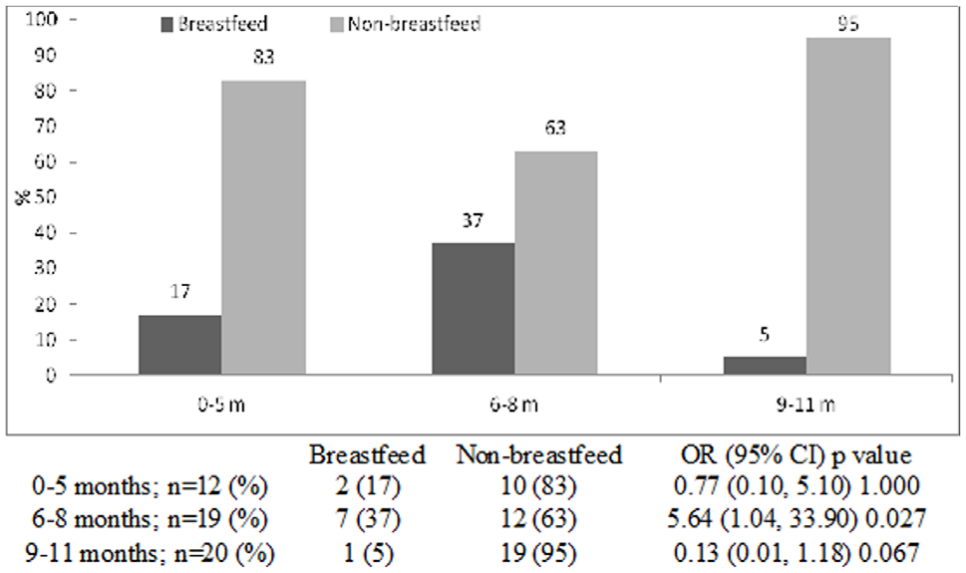
Age stratified proportion of *Shigella* among breastfed and non-breastfed infants presented in the Dhaka Hospital, 2008–11.

The proportion of breast-fed infants gradually fell from 31% in 2008 to 17% in 2011, with gradual increase in the proportion of non-breast-fed infants from 69% to 83% during the same time period (chi square for trend <0.001) (Figure 1). Rotavirus was identified from 43% of breastfed infants aged 0–5 months which gradually reduced to 18% in 9–11 months old; while among the non-breast-fed the proportion infected with this pathogen was 57% in the 0–5 months age groups which increased to to 82% among 9–11 months (chi square for trend <0.001) (Figure 2). Similar observations were made for cholera and ETEC diarrheas among infants (Figure 3 and 4). Although the proportion of breast-fed infants with shigellosis increased to 37% at 6–8 months which was 17% at 0–5 months old, an insignificant rising trend with increasing age was observed among non-breastfed infants (chi square for trend 0.241) (Figure 5). Age stratified proportion of diarrhea pathogens among the breastfed and non-breastfed infants and yearly distribution of different diarrhea pathogens among the breastfed infants have also been shown in table 1 and 2 respectively as the summary results.

**Table 1:** Age stratified proportion of diarrhea pathogens among the breastfed and non-breastfed infants.

	Breastfeedn = 756(%)	Non-breastfeedn = 2113(%)	OR (95% CI); p value
0–5 months	192 (25)	258 (12)	2.45 (1.97, 3.03); <0.001
6–8 months	149 (20)	558 (26)	0.68 (0.56, 0.84); <0.001
9–11 months	116 (15)	528 (25)	0.54 (0.43, 0.63); <0.001

Figures represent n (%) unless indicated otherwise.

**Table 2:** Yearly distribution of diarrhea pathogens among breastfed infants.

Name of the pathogens	2008n = 214(%)	2009n = 190(%)	2010n = 221(%)	2011n = 131(%)
Rotavirus	110 (52)	96 (51)	93 (43)	53 (41)
*Vibrio cholerae* O1	6 (3)	4 (2)	6 (3)	0
ETEC	35 (16)	15 (8)	15 (7)	14 (11)
*Shigella*	5 (2)	2 (1)	1 (0.5)	2 (2)

Figures represent n (%) unless indicated otherwise.

## Discussion

We noted lesser proportion of hospital visits by breastfed infants infected with all the enteric pathogens we analyzed than non-breastfed infants. Breast milk contains non-inflammatogenic secretory immunoglobulin A (sIgA) antibodies that protects intestinal mucosa against the attack from *Vibrio cholerae*, ETEC, *Campylobacter,* and *Shigella* [11], [20], [21], [22], which explains our findings. Literature suggests protective role of breastfeeding against shigellosis and cholera among infants older than 6 months. [8]. However, the breastfed compared to non-breastfed 0–5 months age groups infected with *Shigella* failed to gain statistical significance might be due to small sample size. Our observation of higher proportion of non-breastfed infants with rotavirus is also consistent with earlier observation [23]. The proportions were similar at the beginning but that changed later because of attrition of breastfeeding rate. A Bangladeshi study among infants reported high levels of neutralizing activity among only 20% of the breast milks and low protective efficacy of breast milks against rotavirus strains [24], another well designed case-control study from the same country reported significant protective role of breast milk during the infancy [25]. In another well designed epidemiologic study among Egyptian infants with rotavirus diarrhea, breastfeeding has been observed to be associated with a lower incidence of rotavirus diarrhea and recommended the promotion of breast-feeding along with improvements in water and sanitation for reducing rotavirus diarrhea [26]. We also noted protective role of breastfeeding in infants older than 5 months. However, the protective role in case of shigellosis at 6–8 months was less pronounced among breastfed and this could be due to more virulence of the organism, low infective dose, and lack of immune-competency among infants of that age group. As *Shigella* is the most virulent organism among the all organisms that have been studied here and consequently acquiring immunity against *Shigella* even during continuation of breast milk sometimes may take longer period and our observation of statistical insignificant difference of proportion of *Shigella* isolates in infants between 6–8 months of age among the breast-fed and non-breast-fed groups might also be due to the same reason [22], [27], [28].

The overall results from the study tell us that the proportion of breast-fed infants with diarrhea is reducing, while the national data gathered from the general population without diarrhoea suggests an increase in prevalence of exclusive breastfeeding among infants. Thus the data indicates lack of protection from breast milk against diarrhoeal disease among the non-breastfed infants. However, the population who reported to the facility was the special population with disease condition resulting from the lack of adherence to the breast feeding which had a potential role for this observation.

The main limitation of the study is that study observations are based on infants who attended the hospital and thus our data may not represent the general population because infants with less severe disease less often seek care from hospital facilities. However, systematic sampling of patients including infants and large data set are among strengths of our analyses. Moreover, despite variations, some differences were not statistically significant possibly due to lack of power with small sample size.

In summary, the present study documented the proportion of breastfed infants significantly decreased over the period.The study finding transparently produces evidence of the protective role of breastfeeding against the most common enteropathogens that cause diarrhea compared to those who were non-breastfed. So, there is a large scale need to give more emphasis on breastfeeding promotion campaigning as well as strengthen the current resources and infrastructures for breastfeeding counseling programs. At the same time, a cohort study in different facilities may be conducted to examine if the results of our study are similar in cases of children presenting with other non-diarrheal but infectious diseases.
